# Association between telomere length and idiopathic normal pressure hydrocephalus: a Mendelian randomization study

**DOI:** 10.3389/fneur.2024.1393825

**Published:** 2024-12-17

**Authors:** Feng Yang, Hanlin Cai, Yimeng Ren, Keru Huang, Hui Gao, Linyuan Qin, Ruihan Wang, Yongping Chen, Liangxue Zhou, Dong Zhou, Qin Chen

**Affiliations:** ^1^Department of Neurology, West China Hospital of Sichuan University, Chengdu, China; ^2^Department of Neurosurgery, West China Hospital of Sichuan University, Chengdu, China

**Keywords:** telomere length, idiopathic normal pressure hydrocephalus, Mendelian randomization, vascular risk factors, causal relationship

## Abstract

**Objective:**

Idiopathic normal pressure hydrocephalus (iNPH) is highly prevalent among elderly individuals, and there is a strong correlation between telomere length and biological aging. However, there is limited evidence to elucidate the relationship between telomere length and iNPH. This study aimed to investigate the associations between telomere length and iNPH using the Mendelian randomization (MR) method.

**Methods:**

The genetic variants of telomere length were obtained from 472,174 UK Biobank individuals. Summary level data of iNPH were acquired from 218,365 individuals of the FinnGen consortium. Five MR estimation methods, including inverse-variance weighting (IVW), MR-Egger regression, weighted median, weighted mode and simple mode, were used for causal inference. Comprehensive sensitivity analyses were conducted to test the robustness of the results. In addition, multivariable MR was further implemented to identify potential mechanisms in the causal pathway from telomere length to iNPH.

**Results:**

Genetically determined longer telomere length was significantly associated with decreased risk of iNPH (OR = 0.44, 95% CI 0.24–0.80; *p* = 0.008). No evident heterogeneity (Cochran *Q* = 138.11, *p* = 0.386) and pleiotropy (MR Egger intercept = 0.01, *p* = 0.514) were observed in the sensitivity analysis. In addition, multivariable MR indicated that the observed association was attenuated after adjustment for several vascular risk factors, including essential hypertension (IVW OR = 0.55, 95% CI 0.30–1.03; *p* = 0.061), type 2 diabetes (IVW OR = 0.71, 95% CI 0.09–5.39; *p* = 0.740) and coronary artery disease (IVW OR = 0.58, 95% CI 0.31–1.07; *p* = 0.082).

**Conclusion:**

Our MR study revealed a strong negative correlation of telomere length with iNPH. The causal relationship might be driven by several vascular risk factors.

## 1 Introduction

Idiopathic normal pressure hydrocephalus (iNPH) is a clinical syndrome characterized by the classical triad of cognitive decline, gait disturbance and urinary incontinence (1). With the aging population, there is a growing research focus on iNPH as a reversible form of dementia. However, until now, the pathophysiology of iNPH has remained largely unknown (2). The existing evidence indicated that age may be a crucial factor in the development of iNPH (3). Epidemiological studies have shown that iNPH primarily occurred in individuals aged sixty and above, and the prevalence of iNPH expanded markedly with age (4). In addition, the elderly were more susceptible to disruptions of cerebrospinal fluid (CSF) dynamics due to the age-related changes in physiology and anatomy (5). Such changes may include brain parenchymal atrophy, arterial sclerosis and impairment in lymphatic drainage, which ultimately result in the occurrence of iNPH (6, 7).

Telomeres are repeat segments at the ends of chromosomes that play a pivotal role in genomic stability (8). With each cell division, telomeres progressively shorten and present a dynamic relationship with the aging process (9). As a result, telomere length has been proposed as a potential marker of biological aging (10). Previous studies have linked telomere length to various aging and aging-related disorders, including cardiovascular diseases, cancers and neurodegenerative diseases (11). Some studies indicated that shorter telomere length was related to increased risk of cardiovascular diseases (12). Longer telomere length seemed to be a strong protective factor in Alzheimer’s disease and Parkinson’s disease (13). In addition, a previous neuroimaging study also demonstrated that shortened telomere length might be related to enlarged lateral ventricle volume (14). Considering a potential link between aging and iNPH, we hypothesized that there might be a connection between telomere length and iNPH. In addition, whether this correlation was causally related deserved systematical assessment.

Mendelian randomization (MR) is a statistical method by which shared genetic and causal relationships can be estimated between exposure of interest and disease outcome (15). Besides, compared with classical epidemiology, the use of genetics can be less susceptible to reverse causation and confounding factors since random allocation occurred at meiosis independent of environmental variables (16). Therefore, we performed a MR study using publicly available genome-wide association study (GWAS) datasets to evaluate the causal association between genetically proxied telomere length and the risk of iNPH. In addition, high prevalence of vascular comorbidities was also frequently observed in patients with iNPH, including hypertension, type 2 diabetes mellitus (T2DM), coronary artery disease (CAD), obesity and hyperlipemia (17, 18). Potential roles of vascular risk factors were further investigated in the causal pathway from telomere length to iNPH using the multivariable MR method.

## 2 Methods

### 2.1 Study design

**Figure 1:**
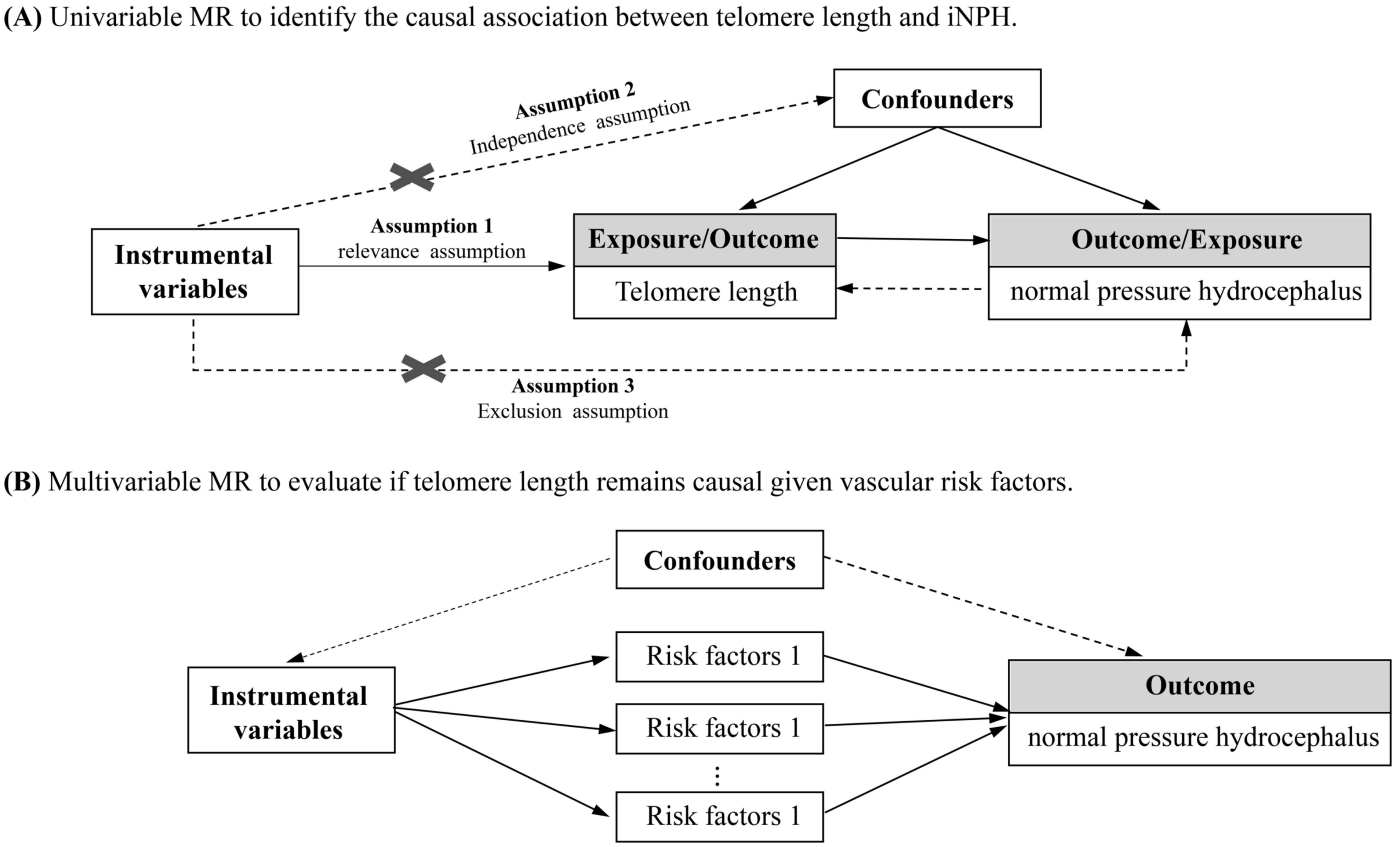
Overview of the study design on the association of telomere length with iNPH. **(A)** Univariable MR to identify the causal association between telomere length and iNPH. **(B)** Multivariable MR to evaluate if telomere length remains causal given vascular risk factors.

In this study, we performed a two-sample MR analysis to investigate the causal relationships between telomere length and iNPH (Figure 1). Firstly, univariable MR (UVMR) was performed to estimate the total causal effect of telomere length on iNPH. In addition, as the direction of causality could be sometimes confusing, a bidirectional MR was applied to avoid the effect of reverse causation (19). Then, multivariable MR (MVMR) was further conducted to investigate the possible mediating role of vascular risk factors in the causal pathway from a genetic perspective (20).

### 2.2 Selection of instrument variants and retrieval of outcome data

We obtained the largest genome-wide association study (GWAS) data of telomere length from Codd et al. (21), which measured 472,174 UK Biobank participants of European ancestry. Telomere length was measured using a well-established quantitative PCR method and adjusted for technical factors, age, gender and ethnicity (22). Outcome iNPH GWAS data were acquired from the FinnGen consortium (23). The diagnosis of NPH was based on the International Classification of Disease 10th Version (ICD-10). Finally, 322 iNPH cases compared with 218,043 controls were involved in the GWAS meta-analysis.

For selecting potential eligible instrumental variants (IVs), the following two criteria were applied: (1) be genome-wide significant association with exposure (*p* < 5 × 10^−8^). If no SNPs met the level, a lower threshold of *p* < 1 × 10–5 was used; (2) be in a threshold of linkage disequilibrium (LD, *r*^2^ < 0.001) within the window size of 10,000 kb. Then, to avoid possible violation of MR key assumptions, we searched for these independent genome-wide significant SNPs in the Phenoscanner database^1^ to exclude pleiotropic genetic instruments from MR analyses. The F-statistic for each SNP was calculated to test their strength (Supplementary Tables S1, S2).

### 2.3 MR analysis

The summary statistics of instrumental variants were then extracted from the outcome datasets. A proxy variant in high LD (*r*^2^ > 0.80) would be searched in the online platform LDlink^2^ if target SNPs were not available in the outcome. However, if no proxies could be identified, the genetic variant would be excluded from the MR analysis.

For UVMR analysis, random effects inverse variance weighting (IVW) was used as the main MR method to estimate the effect of telomere length on iNPH (24). Four additional MR methods were implemented as pleiotropy-robust complements, including MR-Egger regression, weighted median, simple mode and weighted mode (24). Furthermore, to avoid any violation of the MR assumptions, comprehensive sensitivity analysis was conducted. First, Cochran’s Q statistic and funnel plots were applied to detect heterogeneity across variant-specific causal effects (25). Second, leave-one-out (LOO) analysis was performed to determine whether the overall IVW estimate was driven by any single SNP (26). The Mendelian randomization pleiotropy residual sum and outlier (MR-PRESSO) method was further utilized to identify possible outliers and make comparisons between raw and outlier-removal MR estimates (25). Third, possible horizontal pleiotropy was indicated by a nonzero MR-Egger intercept (27). Fourth, different *p*-value thresholds for instrument selection were established to validate the robustness of the findings, including 5e-10, 5e-09, and 5e-07.

For MVMR analysis, considering the fact that with age increasing, there is a corresponding escalation in the prevalence of vascular risk factors. We adjusted for these confounding factors to assess their potential interference on causal effect, including essential hypertension, type 2 diabetes mellitus (T2DM), coronary artery disease (CAD), body mass index (BMI), triglycerides, high-density lipoprotein cholesterol (HDL-C) and low-density lipoprotein cholesterol (LDL-C). MVMR is an extension of UVMR that allows detecting causal effects of multiple risk factors jointly (27). The SNPs used to conduct MVMR were combinations of IVs of each exposure. Similarly, significant (*p* < 5 × 10^−8^) and independent (*r*^2^ < 0.001) criteria were applied to select potential eligible IVs. The sources of GWAS summary data for these traits were listed in Table 1. IVW, weighted median and MR-Egger regression were employed in the MVMR analysis. Heterogeneity of the IVW method was evaluated based on the Q-statistic, and the presence of residual pleiotropy was detected by a multivariable MR-Egger intercept test. Further, we also performed UVMR analysis to investigate the causal effect of genetic liability for telomere length on the possible observed risk factors for iNPH.

**Table 1:** Detailed information of datasets in the current study.

Phenotypes	Unit	Consortium	Ancestry	Sample size	GWAS ID
Telomere length	NA	UK biobank	European	202,046	ieu-b-4879
NPH	NA	FinnGen consortium	European	218,365	finn-b-G6_HCNP
Essential hypertension	SD	MRC-IEU	European	463,010	ukb-b-12493
Type 2 diabetes	NA	NA	European	298,957	ebi-a-GCST007515
Coronary artery disease	log OR	NA	European	547,261	ebi-a-GCST005195
Body mass index	SD	UK biobank	European	461,460	ukb-b-19953
Triglycerides	SD	MRC-IEU	European	441,016	ieu-b-111
HDL-C	SD	MRC-IEU	European	403,947	ieu-b-109
LDL-C	SD	MRC-IEU	European	440,546	ieu-b-110

NPH, normal pressure hydrocephalus; HDL-C, high-density lipoprotein cholesterol; LDL-C, low-density lipoprotein cholesterol.

All statistical analyses were performed using the R packages “TwoSample MR (version 0.5.6)” and “MendelianRandomization (version 0.9.0)” via R (version 4.2.2). For the binary outcome variables, odds ratios (ORs) with corresponding 95% confidence intervals (CIs) were applied to quantify the risk of MR estimates. A *p* value below 0.05 was considered as statistically significant. No additional ethical approval was required for this study.

## 3 Results

### 3.1 UVMR analysis

After data harmonization, the number of instrumental variables was 137 for the causal inference from telomere length to normal pressure hydrocephalus and 27 in a reverse direction. The F statistics of all the SNPs were above 10, indicating the strong strength of instrumental variants (Supplementary Table S1).

**Figure 2:**
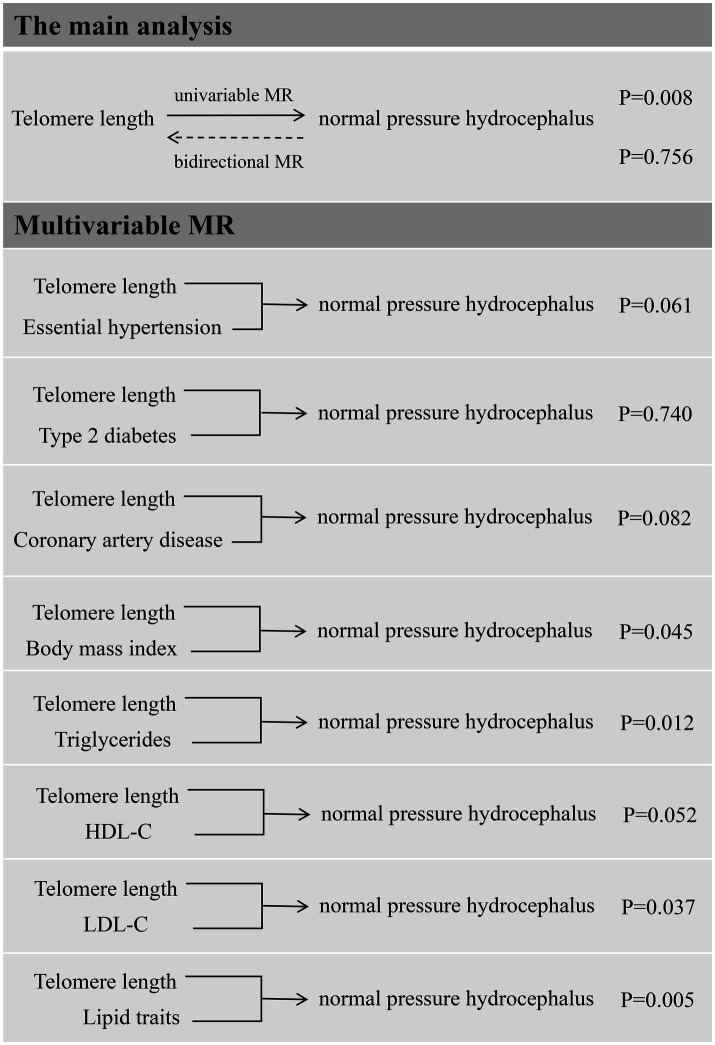
Overview of the main results of Mendelian randomization analysis.

**Figure 3:**
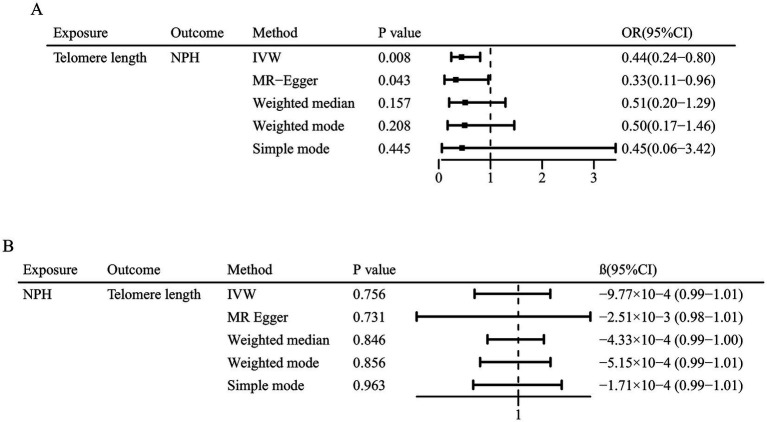
Mendelian randomization associations of **(A)** genetic predicted telomere length on normal pressure hydrocephalus and **(B)** genetic predicted normal pressure hydrocephalus on telomere length.

The main results are summarized in Figure 2. In the univariable MR analysis, the IVW method estimate indicated that genetically determined longer telomere length significantly decreased the risk of iNPH (OR = 0.44; 95% CI 0.24–0.80; *p* = 0.008). Consistent results were detected in the MR-Egger method (OR = 0.33; 95% CI 0.11–0.96; *p* = 0.043), but less significant in the weighted median method (OR = 0.51; 95% CI 0.20–1.29; *p* = 0.157), simple mode (OR = 0.45; 95% CI 0.06–3.42; *p* = 0.445) and weighted mode method (OR = 0.50; 95% CI 0.17–1.46; *p* = 0.208) (Figure 3A). The results of the sensitivity analysis indicated that there was no heterogeneity in the causal effect between telomere length and iNPH (*Q* = 138.11, *p* = 0.386) and the funnel plot was symmetrical (Supplementary Figure S1A). Meanwhile, the LOO analysis revealed that no SNPs drove the results (Supplementary Figure S1B) and there was no evidence of pleiotropy in the MR-Egger regression (intercept = 0.01, *p* = 0.514). Moreover, MR-PRESSO identified no outlier remaining to be removed, supporting the robustness of the MR results. The MR results using different *p*-value thresholds for instrument selection revealed comparable and significant odds ratios (Supplementary Table S3).

In reverse MR analysis, IVW method indicated no causal effect of normal pressure hydrocephalus on telomere length (*β* = −9.77 × 10^−4^; 95% CI 0.99–1.01; *p* = 0.756). Similarly, the other four MR methods suggested insignificant association between them (Figure 3B).

### 3.2 MVMR analysis

**Figure 4:**
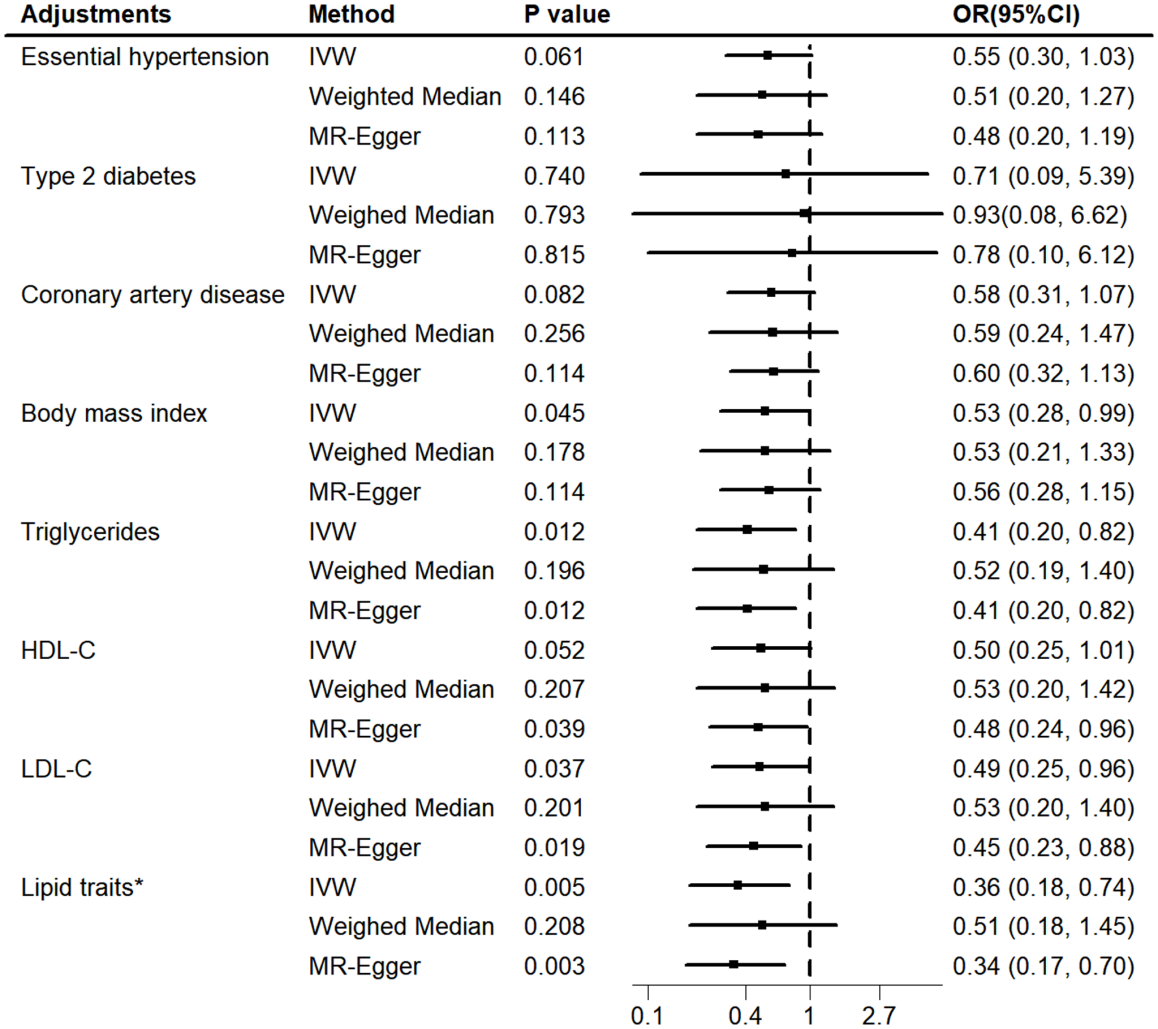
MVMR adjusted for vascular risk factors. MVMR, multivariable Mendelian randomization; HDL-C, high-density lipoprotein cholesterol. LDL-C, low-density lipoprotein cholesterol. Lipid traits* included triglycerides, HDL-C and LDL-C.

Using the MVMR approach, we examined whether the observed association was driven by potential vascular risk factors (Figure 4; Supplementary Table S4). The causal effect of genetically determined telomere length on iNPH remained after adjusting for BMI (IVW OR = 0.53, 95% CI 0.28–0.99; *p* = 0.045), triglycerides (IVW OR = 0.41, 95% CI 0.20–0.82; *p* = 0.012) and LDL-C (IVW OR = 0.49, 95% CI 0.25–0.96; *p* = 0.037). However, the relationship was attenuated after accounting for essential hypertension (IVW OR = 0.55, 95% CI 0.30–1.03; *p* = 0.061), type 2 diabetes (IVW OR = 0.71, 95% CI 0.09–5.39; *p* = 0.740), coronary artery disease (IVW OR = 0.58, 95% CI 0.31–1.07; *p* = 0.082) and HDL-C (IVW OR = 0.50, 95% CI 0.25–1.01; *p* = 0.052). In addition, as lipid-related exposures were genetically and phenotypically correlated (25), we further simultaneously adjusted for triglycerides, HDL-C and LDL-C traits in a model. The causal relationship between telomere length and iNPH remained after accounting for lipid fractions (IVW OR = 0.36, 95% CI 0.18–0.74; *p* = 0.005). No obvious heterogeneities were observed in the Q test analysis in the models. MR-Egger intercept tests showed no evidence of horizontal pleiotropy except for that of LDL-C risk factors. To explore the mechanisms further, additional MR analyses were conducted. The results showed that telomere length was negatively associated with coronary artery disease (IVW OR = 0.84, 95% CI 0.78–0.90; *p*<0.001) and positively associated with essential hypertension (IVW OR = 1.01, 95% CI 1.01–1.02; *p*<0.001), while no association was found for type 2 diabetes (IVW OR = 0.94, 95% CI 0.68–1.30; *p* = 0.710) (Supplementary Table S5).

## 4 Discussion

By using extensive GWAS data for genetic proxies, our results revealed a strong negative correlation of telomere length with iNPH using multiple MR methods. In addition, MVMR analysis indicated that the observed causality might be driven by several vascular risk factors. To our knowledge, this is the first MR study to evaluate the causal effect of telomere length on iNPH and to explore potential mechanisms in the causal pathway.

Genetic liability to shorter telomere length in this study was found to have a total effect on the risk of iNPH. The mechanism of the relationship remains elusive. One possible mechanism may be related to aging-related processes, as indicated by shortened telomeres. Firstly, pulsatile CSF flow may increase due to age-related changes of reduced arterial pulsatility and intracranial compliance (28). As a result, the pressure gradient within and outside the ventricles elevate significantly, ultimately resulting in ventricular dilation (29). Secondly, the brain’s ability to reabsorb CSF may decline with age, leading to inappropriate accumulation of fluid in the brain’s ventricles (30). As a result of age-related decline in intracranial compliance, there could be a more rigid CSF circulatory channels and elevated venous pressure, thus contributing to CSF malabsorption through the arachnoid villi (31). In addition, disruptions in lymphatic pathways are common in both aged mice and humans (32, 33). The disturbance of the glmphatic system can potentially result in the buildup of neurotoxic substances (34), such as beta amyloid (Aβ) and hyperphosphorylated tau (Hp tau), which are thought to be related to cognitive decline in iNPH (35). Beyond aging-related processes, previous studies have reported that accelerated telomere shortening was associated with chronic inflammation, which may contribute to the pathophysiology of iNPH (36). Elevated levels of inflammatory markers have been identified in the CSF of patients with iNPH, including CCL28, CL23, CCL4, CCL3, IL-8 and MCP-1 (37, 38). These markers are associated with the activation of multiple cell types in the choroid plexus and contribute to dysfunction of the blood brain barrier (39, 40). In addition, the present study cannot exclude that the correlation between telomere length and iNPH is influenced by other aging biomarkers (e.g., mitochondrial dysfunction). Further studies are needed to explore the mechanistic pathway from telomere length to iNPH.

Our MVMR analyses indicated that the causal effect of telomere length on iNPH could be attributed, at least partially to the effects of certain vascular risk factors. The effect of telomere length on iNPH was significantly attenuated after adjusting for essential hypertension, T2DM and CAD. With increasing age, there is an inevitable escalation in the prevalence of vascular risk factors (41). Indeed, the genetic variants for telomere length measure the lifetime attrition of telomeres and might be indicative of certain underlying biological conditions that could lead to iNPH (42). In this context, it was possible that essential hypertension, T2DM and CAD ultimately contributed to the incidence of iNPH rather than telomere length *per se*. Previous studies have reported extensive linkage between vascular risk factors and iNPH (43). Among these factors, hypertension was found to be the most common vascular comorbidity in iNPH (17, 18). A systematic literature review has revealed that the occurrence rate of diabetes in iNPH patients was more than twice as frequent as that in age-matched controls (43). In addition, the association between CAD and iNPH was also observed in some studies, with overrepresented vasculopathy in both magnetic resonance imaging and autopsy-based findings (44, 45).

However, in our additional analyses of risk factors for iNPH, we found that telomere length was associated with an increased risk of essential hypertension and showed no association with T2DM, suggesting that hypertension and T2DM may be confounding factors. Meanwhile, we found that telomere length was strongly associated with a reduced risk of CAD, which might mediate the decreased risk of iNPH. Therefore, we need to pay close attention to the elderly population with CAD. Indeed, MVMR has limitations in establishing mediation due to its reliance on strong assumptions (46). As it is impossible to completely distinguish mediation from the pleiotropic effects of the SNPs (47), we pooled all potentially relevant phenotypes for the instrumental variables of telomere length in Supplementary Table S2. Future studies may need to rigorously control for vascular risk factors such as essential hypertension, T2DM, and CAD in other cohorts, or conduct stratified analyses to reveal the true relationship between telomere length and iNPH.

Our study has several strengths. First, we excluded genetic variants associated with potential confounders commonly found in epidemiological studies and selected only the SNPs that are strongly linked to telomere length (48, 49). Second, multiple methods of MR were utilized to minimize biased estimates and facilitate the identification of consistent results, including MR-Egger regression, weighted median, simple mode and weighted mode (50). Third, a series of sensitivity analyses were conducted to control for pleiotropic bias and validate the robustness of the MR results, such as the Cochran’s Q statistic, funnel plots and leave-one-out analysis (51). Based on the methodological strengths of this study, the findings may provide clues for the development of telomere length as a novel biomarker for early screening of people at risk for iNPH and further exploration of possible therapeutic strategies targeting telomere dynamics.

Despite the strengths, the study had limitations. First, there are limited GWAS data for NPH available. The GWAS data for iNPH is relatively small, which may limit statistical power and the ability to detect weaker associations. Future research is necessary to validate our results when larger GWAS datasets become available. In addition, we could not exclude the presence of secondary NPH cases in the outcome. Therefore, when focusing on iNPH, potentially biased results should be acknowledged. Second, the participants in the study were all of European ancestry. While this may help avoid population heterogeneity, it also limits the generalizability of the findings. Additional investigation is needed to extrapolate the findings to other populations. Third, while our sensitivity analyses incorporating complementary MR methods failed to find evidence of pleiotropy, it is still possible that pleiotropy may be present in our study (for example, the SNPs related to telomere length may affect iNPH through pathways other than vascular risk factors), as 44% of genes are linked to more than one phenotype in humans and the number of phenotypes associated with each gene ranges from 1 to 53 (52, 53). Fourth, although the study provided preliminary genetic evidence supporting a protective effect of telomere length on iNPH, we should interpret the results with caution. As telomere length was assessed with genetic variants, which are inheritable and fixed at conception, rather than actual measures of exposures and outcomes, further clinical trials in real-world settings with large sample sizes are necessary to provide definitive evidence.

## 5 Conclusion

In conclusion, our MR study supports the causal effect of telomere shortening on risk of iNPH. In addition, essential hypertension, T2DM and CAD might act in the telomere length-iNPH pathway, while there was no enough evidence for other vascular risk factors in the causality. These findings help us better understand the pathophysiology of iNPH and highlight the role of vascular risk factors underlying the causal association. More research is needed to confirm the findings and to explain the cellular and molecular mechanisms behind them.

## Data Availability

The original contributions presented in the study are included in the article/Supplementary material, further inquiries can be directed to the corresponding author.

## Footnotes

^1^ http://www.phenoscanner.medschl.cam.ac.uk/

^2^ https://ldlink.nci.nih.gov/
