# Supplementary material for: Association between telomere length and idiopathic normal pressure hydrocephalus: a Mendelian randomization study
Source: Front Neurol. 2024 Dec 17;15:1393825. doi: 10.3389/fneur.2024.1393825 (PMC11686450; doi:10.3389/fneur.2024.1393825)
Supplement: Supplementary file 1 [file Table_1.docx]

Supplementary Material

**Association between telomere length and idiopathic normal pressure hydrocephalus: a Mendelian randomization study**

**Feng Yang^1†^, Hanlin Cai^1†^, Yimeng Ren^1^, Keru Huang^2^, Hui Gao^1^, Linyuan Qin^1^, Ruihan Wang^1^, Yongping Chen^1^, Liangxue Zhou^2^, Dong Zhou^1^, Qin Chen^1*^**

*** Correspondence:** Qin Chen, chen.qin@scu.edu.cn

**List of supplementary materials**

| **Code** | **Material** |
| --- | --- |
| Table S1 | Instrumental variables of telomere length included in the Mendelian randomization analysis. |
| Table S2 | All associated Traits of telomere length SNPs on PhenoScanner. |
| Table S3 | MR results using different p-value thresholds for instrument selection. |
| Table S4 | Complete MVMR results in current study. |
| Table S5 | Univariable MR results for the effects of telomere length on vascular risk factors. |
| Table S6 | Percentage of comorbid patients in normal pressure hydrocephalus (NPH) cases |
| Figure S1 | Sensitivity analysis for the association between telomere length and iNPH. **(A)** Funnel plot **(B)** Leave-one-out analysis |

**Table S1.** Instrumental variables of telomere length included in the Mendelian randomization analysis.

| SNP | EA | OA | Eaf | Beta | SE | P | R2 | F |
| --- | --- | --- | --- | --- | --- | --- | --- | --- |
| rs1003322 | A | C | 0.2137 | 0.0142 | 0.0025 | 1.00E-08 | 0.0001 | 31.88 |
| rs10112752 | A | G | 0.4304 | -0.0288 | 0.0020 | 9.50E-46 | 0.0004 | 191.46 |
| rs1023767 | A | G | 0.2376 | -0.0184 | 0.0023 | 5.00E-15 | 0.0001 | 57.75 |
| rs10768683 | G | C | 0.8410 | 0.0470 | 0.0028 | 1.50E-64 | 0.0006 | 278.97 |
| rs10773176 | G | A | 0.7412 | -0.0172 | 0.0023 | 5.20E-14 | 0.0001 | 53.60 |
| rs10774624 | A | G | 0.5328 | 0.0150 | 0.0021 | 2.90E-13 | 0.0001 | 52.86 |
| rs10805346 | C | T | 0.4393 | 0.0117 | 0.0020 | 7.00E-09 | 0.0001 | 31.88 |
| rs10840270 | G | C | 0.6557 | 0.0144 | 0.0021 | 1.30E-11 | 0.0001 | 44.11 |
| rs10845387 | A | G | 0.3527 | -0.0141 | 0.0021 | 1.50E-11 | 0.0001 | 42.99 |
| rs10905255 | T | G | 0.5792 | -0.0182 | 0.0020 | 2.60E-19 | 0.0002 | 76.67 |
| rs11085072 | T | C | 0.2369 | -0.0132 | 0.0024 | 2.60E-08 | 0.0001 | 29.66 |
| rs11117354 | C | T | 0.6965 | 0.0233 | 0.0022 | 3.40E-26 | 0.0002 | 107.94 |
| rs111527438 | C | T | 0.3513 | 0.0125 | 0.0021 | 3.10E-09 | 0.0001 | 33.63 |
| rs111950327 | C | G | 0.0636 | 0.0238 | 0.0041 | 5.90E-09 | 0.0001 | 31.94 |
| rs11212631 | C | T | 0.1992 | -0.0193 | 0.0026 | 4.70E-14 | 0.0001 | 56.39 |
| rs112394943 | C | T | 0.1627 | -0.0199 | 0.0028 | 1.60E-12 | 0.0001 | 50.94 |
| rs113525195 | A | C | 0.2903 | -0.0124 | 0.0022 | 3.10E-08 | 0.0001 | 29.95 |
| rs11412296 | T | TA | 0.7594 | 0.0332 | 0.0023 | 1.40E-45 | 0.0004 | 190.57 |
| rs11426156 | T | TA | 0.3995 | -0.0116 | 0.0021 | 2.20E-08 | 0.0001 | 30.42 |
| rs11557154 | T | C | 0.1300 | -0.0344 | 0.0030 | 1.10E-30 | 0.0003 | 126.24 |
| rs11579626 | C | A | 0.0849 | 0.0265 | 0.0036 | 1.30E-13 | 0.0001 | 51.56 |
| rs11584821 | T | C | 0.1762 | -0.0307 | 0.0026 | 3.00E-31 | 0.0003 | 128.83 |
| rs116863223 | A | G | 0.0118 | -0.0818 | 0.0094 | 2.60E-18 | 0.0002 | 73.44 |
| rs11699829 | A | G | 0.0341 | 0.0642 | 0.0060 | 1.50E-26 | 0.0003 | 128.38 |
| rs117034449 | A | G | 0.0233 | 0.0374 | 0.0067 | 2.10E-08 | 0.0001 | 30.13 |
| rs117407747 | T | C | 0.0276 | 0.0451 | 0.0061 | 1.80E-13 | 0.0001 | 51.40 |
| rs117512405 | A | G | 0.0170 | -0.0790 | 0.0082 | 9.50E-22 | 0.0002 | 98.79 |
| rs117630647 | A | G | 0.0213 | 0.0596 | 0.0072 | 1.40E-16 | 0.0001 | 70.00 |
| rs11769630 | A | T | 0.0722 | -0.0257 | 0.0039 | 4.30E-11 | 0.0001 | 41.74 |
| rs11991877 | A | T | 0.8893 | -0.0301 | 0.0032 | 3.20E-21 | 0.0002 | 84.45 |
| rs12369950 | C | T | 0.1407 | -0.0178 | 0.0029 | 8.00E-10 | 0.0001 | 36.30 |
| rs12412214 | A | G | 0.2798 | -0.0245 | 0.0022 | 3.40E-28 | 0.0002 | 114.41 |
| rs12451892 | C | T | 0.3805 | -0.0116 | 0.0021 | 2.20E-08 | 0.0001 | 30.03 |
| rs1291143 | C | A | 0.8490 | 0.0493 | 0.0028 | 1.80E-69 | 0.0006 | 294.56 |
| rs12925933 | C | A | 0.6622 | -0.0147 | 0.0021 | 7.00E-12 | 0.0001 | 45.42 |
| rs12932179 | G | A | 0.5614 | -0.0136 | 0.0020 | 1.80E-11 | 0.0001 | 43.17 |
| rs13062095 | C | T | 0.3278 | 0.0139 | 0.0021 | 9.70E-11 | 0.0001 | 39.95 |
| rs131797 | T | TAAAAA | 0.2356 | 0.0244 | 0.0024 | 6.80E-25 | 0.0002 | 101.08 |
| rs13230646 | C | T | 0.2489 | -0.0173 | 0.0023 | 8.90E-14 | 0.0001 | 53.02 |
| rs1332941 | G | A | 0.8205 | 0.0257 | 0.0027 | 5.90E-21 | 0.0002 | 91.57 |
| rs137901416 | A | G | 0.1003 | 0.0457 | 0.0033 | 4.70E-43 | 0.0004 | 178.22 |
| rs139669835 | T | C | 0.0094 | -0.0613 | 0.0105 | 6.10E-09 | 0.0001 | 32.88 |
| rs139795227 | C | A | 0.0140 | 0.0599 | 0.0087 | 6.70E-12 | 0.0001 | 46.91 |
| rs141214782 | TTATC | T | 0.1012 | -0.0247 | 0.0034 | 2.00E-13 | 0.0001 | 52.28 |
| rs142426306 | T | C | 0.0395 | -0.0505 | 0.0054 | 8.70E-21 | 0.0002 | 91.45 |
| rs142730696 | TTTTTC | T | 0.8641 | 0.0217 | 0.0030 | 6.30E-13 | 0.0001 | 52.20 |
| rs143190905 | T | G | 0.0804 | -0.0724 | 0.0037 | 1.60E-85 | 0.0008 | 366.28 |
| rs144204502 | T | C | 0.0126 | -0.1006 | 0.0091 | 3.40E-28 | 0.0003 | 118.52 |
| rs145114957 | G | C | 0.0426 | 0.0273 | 0.0050 | 4.60E-08 | 0.0001 | 28.65 |
| rs150150565 | T | C | 0.0215 | 0.0638 | 0.0074 | 6.80E-18 | 0.0002 | 80.62 |
| rs1611236 | A | G | 0.3269 | -0.0160 | 0.0021 | 6.10E-14 | 0.0001 | 53.29 |
| rs16978028 | T | A | 0.1437 | -0.0299 | 0.0029 | 8.20E-26 | 0.0002 | 104.24 |
| rs17445108 | A | G | 0.1270 | -0.0169 | 0.0030 | 2.00E-08 | 0.0001 | 29.87 |
| rs17677991 | G | C | 0.3421 | 0.0223 | 0.0021 | 4.40E-26 | 0.0002 | 105.40 |
| rs17803849 | T | C | 0.4052 | 0.0273 | 0.0020 | 4.20E-41 | 0.0004 | 169.94 |
| rs182059586 | C | T | 0.0251 | -0.0571 | 0.0068 | 4.90E-17 | 0.0002 | 75.43 |
| rs185174247 | A | G | 0.0561 | 0.0373 | 0.0044 | 1.10E-17 | 0.0001 | 69.50 |
| rs188918174 | T | C | 0.0361 | 0.0403 | 0.0054 | 1.20E-13 | 0.0001 | 53.40 |
| rs1907702 | A | G | 0.7668 | 0.0150 | 0.0024 | 5.90E-10 | 0.0001 | 38.13 |
| rs1957937 | T | A | 0.1602 | 0.0209 | 0.0027 | 1.90E-14 | 0.0001 | 55.69 |
| rs1985369 | G | A | 0.8682 | -0.0312 | 0.0030 | 3.60E-25 | 0.0002 | 105.16 |
| rs201558190 | C | T | 0.3633 | -0.0182 | 0.0022 | 6.40E-17 | 0.0002 | 72.16 |
| rs202034370 | T | TA | 0.9754 | 0.1028 | 0.0065 | 2.60E-56 | 0.0005 | 239.41 |
| rs2056726 | A | G | 0.2144 | -0.0228 | 0.0024 | 7.90E-21 | 0.0002 | 82.75 |
| rs2230590 | C | T | 0.5109 | -0.0158 | 0.0020 | 3.60E-15 | 0.0001 | 58.93 |
| rs2276182 | G | C | 0.4032 | 0.0234 | 0.0020 | 2.80E-30 | 0.0003 | 123.96 |
| rs2282764 | G | A | 0.1424 | -0.0224 | 0.0029 | 9.30E-15 | 0.0001 | 57.99 |
| rs2293579 | A | G | 0.3863 | -0.0129 | 0.0021 | 3.30E-10 | 0.0001 | 37.34 |
| rs2306646 | C | G | 0.5595 | -0.0209 | 0.0020 | 3.30E-25 | 0.0002 | 102.09 |
| rs2538745 | C | T | 0.6028 | -0.0129 | 0.0021 | 3.10E-10 | 0.0001 | 37.87 |
| rs2555104 | C | A | 0.4343 | -0.0140 | 0.0020 | 6.60E-12 | 0.0001 | 45.29 |
| rs2763979 | T | C | 0.3597 | -0.0278 | 0.0021 | 1.30E-40 | 0.0004 | 167.81 |
| rs28363070 | A | G | 0.0134 | 0.0756 | 0.0096 | 3.50E-15 | 0.0002 | 71.22 |
| rs28502153 | A | C | 0.3780 | -0.0216 | 0.0021 | 1.20E-25 | 0.0002 | 103.53 |
| rs28577594 | C | G | 0.7098 | 0.0188 | 0.0022 | 5.40E-17 | 0.0001 | 68.51 |
| rs2967355 | C | A | 0.7743 | -0.0462 | 0.0024 | 4.00E-83 | 0.0007 | 351.92 |
| rs2977608 | C | A | 0.7439 | 0.0129 | 0.0023 | 3.00E-08 | 0.0001 | 30.16 |
| rs3093888 | A | G | 0.0513 | -0.0290 | 0.0045 | 1.50E-10 | 0.0001 | 38.59 |
| rs34550383 | C | CT | 0.5474 | -0.0192 | 0.0020 | 8.60E-22 | 0.0002 | 86.48 |
| rs34896435 | G | C | 0.4692 | 0.0155 | 0.0021 | 6.40E-14 | 0.0001 | 56.20 |
| rs35446936 | A | G | 0.2437 | -0.0940 | 0.0023 | 1.00E-200 | 0.0033 | 1542.92 |
| rs35500378 | CACTT | C | 0.6105 | 0.0145 | 0.0021 | 2.00E-12 | 0.0001 | 47.00 |
| rs35640778 | A | G | 0.0208 | -0.2090 | 0.0070 | 9.59E-195 | 0.0018 | 840.03 |
| rs376641875 | C | CATAA | 0.9289 | -0.0266 | 0.0042 | 3.30E-10 | 0.0001 | 44.10 |
| rs3767952 | A | G | 0.2267 | 0.0134 | 0.0024 | 1.80E-08 | 0.0001 | 29.94 |
| rs3785074 | G | A | 0.2897 | 0.0239 | 0.0022 | 2.60E-27 | 0.0002 | 110.67 |
| rs3891167 | G | A | 0.2534 | -0.0426 | 0.0024 | 1.20E-70 | 0.0007 | 324.00 |
| rs41269079 | A | T | 0.1890 | 0.0154 | 0.0025 | 1.70E-09 | 0.0001 | 34.16 |
| rs41304832 | A | G | 0.0124 | 0.0612 | 0.0093 | 5.00E-11 | 0.0001 | 43.20 |
| rs429358 | C | T | 0.1540 | 0.0173 | 0.0028 | 3.80E-10 | 0.0001 | 37.03 |
| rs4498805 | T | G | 0.5466 | 0.0151 | 0.0020 | 5.70E-14 | 0.0001 | 53.09 |
| rs450962 | G | A | 0.2838 | 0.0143 | 0.0025 | 5.90E-09 | 0.0001 | 39.16 |
| rs4530278 | T | G | 0.5982 | 0.0139 | 0.0021 | 1.50E-11 | 0.0001 | 43.73 |
| rs45604339 | T | C | 0.3424 | -0.0204 | 0.0021 | 4.30E-22 | 0.0002 | 88.79 |
| rs4616688 | T | G | 0.5254 | -0.0173 | 0.0020 | 4.50E-18 | 0.0002 | 70.87 |
| rs4695407 | G | A | 0.5078 | 0.0142 | 0.0020 | 1.50E-12 | 0.0001 | 47.27 |
| rs4724 | A | G | 0.1166 | -0.0547 | 0.0031 | 9.80E-69 | 0.0006 | 291.70 |
| rs4731541 | G | C | 0.6249 | -0.0206 | 0.0021 | 1.40E-23 | 0.0002 | 94.06 |
| rs4743037 | T | C | 0.2309 | 0.0148 | 0.0024 | 5.10E-10 | 0.0001 | 36.72 |
| rs55747751 | A | G | 0.0774 | -0.0212 | 0.0038 | 1.70E-08 | 0.0001 | 30.19 |
| rs56061761 | A | G | 0.3326 | -0.0204 | 0.0022 | 6.90E-20 | 0.0002 | 86.88 |
| rs56178008 | A | T | 0.4375 | 0.0144 | 0.0020 | 9.70E-13 | 0.0001 | 48.02 |
| rs56799554 | G | A | 0.1702 | -0.0260 | 0.0027 | 3.00E-22 | 0.0002 | 90.03 |
| rs5742915 | C | T | 0.4458 | 0.0193 | 0.0020 | 1.60E-21 | 0.0002 | 87.26 |
| rs59409453 | G | A | 0.7306 | 0.0202 | 0.0023 | 1.60E-18 | 0.0002 | 75.95 |
| rs6007020 | C | T | 0.3678 | 0.0145 | 0.0021 | 4.80E-12 | 0.0001 | 46.11 |
| rs6054257 | A | G | 0.7935 | -0.0142 | 0.0025 | 1.10E-08 | 0.0001 | 31.06 |
| rs611646 | A | T | 0.4087 | -0.0368 | 0.0020 | 3.50E-73 | 0.0007 | 309.78 |
| rs61405042 | T | C | 0.0293 | -0.0502 | 0.0060 | 8.50E-17 | 0.0001 | 67.63 |
| rs61748181 | T | C | 0.0289 | -0.0592 | 0.0060 | 2.80E-23 | 0.0002 | 92.93 |
| rs6536702 | A | G | 0.7746 | 0.0534 | 0.0024 | 9.40E-111 | 0.0010 | 470.82 |
| rs6584579 | G | A | 0.3989 | 0.0115 | 0.0020 | 2.00E-08 | 0.0001 | 29.91 |
| rs6587577 | G | A | 0.8263 | -0.0182 | 0.0026 | 4.80E-12 | 0.0001 | 44.96 |
| rs6590343 | G | A | 0.5164 | 0.0122 | 0.0020 | 1.50E-09 | 0.0001 | 34.95 |
| rs6659669 | T | C | 0.6051 | -0.0117 | 0.0021 | 1.10E-08 | 0.0001 | 30.94 |
| rs6669563 | A | G | 0.4378 | 0.0182 | 0.0020 | 2.10E-19 | 0.0002 | 77.31 |
| rs66731853 | A | G | 0.3173 | -0.0178 | 0.0022 | 1.50E-16 | 0.0001 | 64.67 |
| rs670180 | A | T | 0.5691 | -0.0116 | 0.0020 | 1.20E-08 | 0.0001 | 31.06 |
| rs6751209 | C | T | 0.2042 | -0.0140 | 0.0025 | 1.60E-08 | 0.0001 | 30.28 |
| rs6776756 | A | G | 0.5976 | -0.0174 | 0.0020 | 1.10E-17 | 0.0001 | 69.11 |
| rs6790988 | G | A | 0.7419 | 0.0146 | 0.0023 | 1.80E-10 | 0.0001 | 38.40 |
| rs6881568 | A | C | 0.3626 | 0.0169 | 0.0021 | 3.70E-16 | 0.0001 | 62.53 |
| rs7099229 | A | G | 0.2733 | -0.0153 | 0.0022 | 8.40E-12 | 0.0001 | 44.07 |
| rs7164950 | G | A | 0.4060 | 0.0129 | 0.0020 | 2.30E-10 | 0.0001 | 38.11 |
| rs7209057 | A | G | 0.5610 | 0.0118 | 0.0020 | 5.70E-09 | 0.0001 | 32.49 |
| rs7221585 | T | C | 0.2240 | 0.0143 | 0.0025 | 6.70E-09 | 0.0001 | 33.70 |
| rs73581419 | T | C | 0.1066 | 0.0230 | 0.0032 | 1.30E-12 | 0.0001 | 47.52 |
| rs73730598 | A | G | 0.0548 | 0.0274 | 0.0044 | 4.70E-10 | 0.0001 | 36.62 |
| rs75664430 | G | C | 0.2480 | -0.0235 | 0.0023 | 3.60E-24 | 0.0002 | 97.44 |
| rs76065543 | T | C | 0.1375 | 0.0343 | 0.0029 | 4.20E-32 | 0.0003 | 131.70 |
| rs76219171 | A | G | 0.0584 | 0.0360 | 0.0043 | 7.80E-17 | 0.0001 | 67.28 |
| rs762679 | A | T | 0.8565 | 0.0310 | 0.0029 | 1.40E-27 | 0.0002 | 111.64 |
| rs76666449 | C | T | 0.1006 | 0.0295 | 0.0033 | 8.20E-19 | 0.0002 | 74.45 |
| rs7705526 | A | C | 0.3266 | 0.0776 | 0.0022 | 1.00E-200 | 0.0026 | 1254.02 |
| rs77231040 | C | G | 0.0057 | 0.0989 | 0.0135 | 2.00E-13 | 0.0001 | 52.77 |
| rs7772289 | T | G | 0.5031 | 0.0175 | 0.0020 | 1.70E-18 | 0.0002 | 72.72 |
| rs77732866 | A | G | 0.1376 | 0.0178 | 0.0029 | 9.20E-10 | 0.0001 | 35.48 |
| rs7790856 | T | C | 0.2891 | -0.0437 | 0.0022 | 1.80E-87 | 0.0008 | 371.30 |
| rs78491606 | C | A | 0.0184 | -0.0756 | 0.0074 | 1.90E-24 | 0.0002 | 97.75 |
| rs79977579 | A | C | 0.0956 | 0.0282 | 0.0034 | 2.30E-16 | 0.0001 | 64.69 |
| rs80116508 | A | G | 0.0624 | -0.0353 | 0.0042 | 2.00E-17 | 0.0001 | 68.68 |
| rs80324517 | A | G | 0.0483 | 0.0397 | 0.0047 | 1.80E-17 | 0.0001 | 68.20 |
| rs8102497 | A | G | 0.4318 | -0.0150 | 0.0020 | 1.40E-13 | 0.0001 | 51.90 |
| rs8105767 | G | A | 0.2947 | 0.0328 | 0.0022 | 2.50E-50 | 0.0004 | 211.75 |
| rs869785 | C | T | 0.6725 | -0.0147 | 0.0021 | 4.40E-12 | 0.0001 | 45.14 |
| rs871134 | T | C | 0.5690 | -0.0183 | 0.0020 | 1.70E-19 | 0.0002 | 77.56 |
| rs932002 | T | C | 0.1508 | -0.0402 | 0.0028 | 7.30E-47 | 0.0004 | 195.61 |
| rs9398196 | G | A | 0.5201 | -0.0144 | 0.0020 | 9.50E-13 | 0.0001 | 48.60 |
| rs939916 | A | G | 0.6700 | 0.0242 | 0.0022 | 6.60E-29 | 0.0003 | 122.11 |
| rs9419958 | C | T | 0.8614 | -0.0810 | 0.0029 | 2.60E-167 | 0.0016 | 741.11 |
| rs9600019 | T | C | 0.3356 | 0.0127 | 0.0021 | 2.40E-09 | 0.0001 | 34.03 |
| rs9878436 | T | C | 0.4344 | -0.0143 | 0.0020 | 1.20E-12 | 0.0001 | 47.72 |
| rs9940099 | T | G | 0.0627 | -0.0336 | 0.0041 | 3.20E-16 | 0.0001 | 62.71 |
| rs9940099 | T | G | 0.0627 | -0.0336 | 0.0041 | 3.20E-16 | 0.0001 | 62.71 |
| rs9955360 | A | C | 0.8693 | -0.0190 | 0.0030 | 2.20E-10 | 0.0001 | 38.87 |

SNP, single nucleotide polymorphism; EA, effect allele; OA, other allele; EAF, effect allele frequency.

**Table S2.** All associated Traits of telomere length SNPs on PhenoScanner. (p-value< 5e-8).

| Code | SNP | Traits |
| --- | --- | --- |
| 1 | rs3767952 | Sitting height |
| 2 | rs4498805 | None |
| 3 | rs932002 | Plateletcrit |
| 4 | rs66731853 | Lymphocyte percentage of white cells, Mean corpuscular volume, Mean corpuscular volume |
| 5 | rs11584821 | None |
| 6 | rs6659669 | None |
| 7 | rs2977608 | None |
| 8 | rs6669563 | None |
| 9 | rs145114957 | None |
| 10 | rs41269079 | None |
| 11 | rs139795227 | Granulocyte percentage of myeloid white cells, Lymphocyte percentage of white cells, Mean corpuscular hemoglobin concentration, Monocyte count, Monocyte percentage of white cells, Myeloid white cell count, Red cell distribution width, White blood cell count, Mouth or teeth dental problems: mouth ulcers |
| 12 | rs11579626 | Height, Comparative height size at age 10, Height, Leg fat-free mass left, Leg predicted mass left |
| 13 | rs6587577 | Impedance of arm left, Impedance of arm right, Impedance of leg right ,Impedance of whole body |
| 14 | rs6751209 | Lymphocyte count, Lymphocyte percentage of white cells, Neutrophil percentage of white cells Platelet count, Plateletcrit, Comparative height size at age 10, Hair or balding pattern: pattern 4 ,Impedance of leg left, Impedance of leg right, Impedance of whole body, Irritability |
| 15 | rs188918174 | None |
| 16 | rs202034370 | None |
| 17 | rs56178008 | Hip circumference |
| 18 | rs376641875 | None |
| 19 | rs17803849 | None |
| 20 | rs2555104 | Illnesses of siblings: none of the above, group 1 |
| 21 | rs77732866 | Body mass index females, Mean corpuscular hemoglobin, Mean corpuscular volume, Red blood cell count |
| 22 | rs2230590 | Age first birth female, Age first birth, Body mass index in males greater than 50 years of age, Body mass index adjusted for smoking, Body mass index adjusted for smoking, Body mass index, Crohns disease, Inflammatory bowel disease, Ulcerative colitis, Age at first live birth, Age at last live birth, Age completed full time education, Alcohol intake frequency, Alcohol intake versus 10 years previously, Arm fat mass left, Arm fat mass right, Arm fat percentage left, Arm fat percentage right, Arm fat-free mass left, Arm fat-free mass right, Arm predicted mass left, Arm predicted mass right, Average weekly beer plus cider intake, Basal metabolic rate, Body fat percentage, Body mass index, Diabetes diagnosed by doctor, Diastolic blood pressure, Ever highly irritable or argumentative for 2 days, Fed-up feelings, Fluid intelligence score, Frequency of stair climbing in last 4 weeks, Heel bone mineral density, Heel bone mineral density left, Hip circumference, Impedance of arm left, Impedance of arm right, Impedance of leg left, Impedance of leg right, Impedance of whole body, Job involves heavy manual or physical work, Job involves mainly walking or standing, Leg fat mass left, Leg fat mass right, Leg fat percentage left, Leg fat percentage right, Leg fat-free mass left, Leg fat-free mass right, Leg pain on walking, Leg predicted mass left, Leg predicted mass right, Miserableness, Mood swings, Mouth or teeth dental problems: dentures, Number of treatments or medications taken, Overall health rating, Pain type experienced in last month: none of the above, Pulse rate, Qualifications: A levels or as levels or equivalent, Qualifications: college or university degree, Qualifications: none, Qualifications: other professional qualifications, Self-reported diabetes, Shoulder lesions, Sleeplessness or insomnia, Taking other prescription medications, Time spent watching television, Treatment with co-codamol, Trunk fat mass, Trunk fat percentage, Trunk fat-free mass, Trunk predicted mass, Types of physical activity in last 4 weeks: other exercises, Types of transport used, excluding work: cycle, Usual walking pace, Waist circumference, Weight, Wheeze or whistling in the chest in last year, Whole body fat mass, Whole body fat-free mass, Whole body water mass, Why stopped smoking: health precaution, Age at menarche, Years of educational attainment in females, Years of educational attainment in males, Years of educational attainment |
| 23 | rs78491606 | Cause of death: aortic valve disorder, unspecified |
| 24 | rs9878436 | Forced vital capacity, Forced vital capacity, best measure, Height, Vascular or heart problems diagnosed by doctor: none of the above ,Coronary artery disease |
| 25 | rs35446936 | Eosinophil count, Eosinophil percentage of granulocytes, Eosinophil percentage of white cells Granulocyte percentage of myeloid white cells, Neutrophil percentage of granulocytes, Neutrophil percentage of white cells, Sum eosinophil basophil counts, Benign neoplasm of colon, rectum, anus and anal canal, Number of operations, Self-reported hypothyroidism or myxoedema ,Treatment with blood pressure medication ,Treatment with levothyroxine sodium |
| 26 | rs112394943 | None |
| 27 | rs13062095 | Eosinophil count ,Eosinophil percentage of granulocytes ,Eosinophil percentage of white cells, Neutrophil percentage of granulocytes, Sum eosinophil basophil counts, Allergic disease, Eosinophil percentage of granulocytes, Eosinophil percentage of white cells, Neutrophil percentage of granulocytes, Arm fat percentage left, Arm fat percentage right, Body fat percentage, Heel bone mineral density, Heel bone mineral density left, Heel bone mineral density right, Height, Leg fat percentage right, Trunk fat percentage |
| 28 | rs4616688 | Eosinophil percentage of granulocytes, Eosinophil percentage of white cells, Neutrophil percentage of granulocytes, Red blood cell count |
| 29 | rs6790988 | None |
| 30 | rs869785 | High light scatter percentage of red cells, High light scatter reticulocyte count, Immature fraction of reticulocytes, Mean corpuscular hemoglobin, Mean corpuscular hemoglobin concentration, Mean corpuscular volume, Monocyte count, Monocyte percentage of white cells, Red blood cell count, Red cell distribution width, Reticulocyte count, Reticulocyte fraction of red cells, Monocyte count |
| 31 | rs6776756 | Basophil count, Lymphocyte percentage of white cells |
| 32 | rs11426156 | Basophil count, Basophil percentage of granulocytes, Basophil percentage of white cells, Eosinophil count, Eosinophil percentage of granulocytes, Eosinophil percentage of white cells, Granulocyte percentage of myeloid white cells, Monocyte count, Monocyte percentage of white cells, Neutrophil percentage of granulocytes, Neutrophil percentage of white cells, Sum eosinophil basophil counts |
| 33 | rs35500378 | None |
| 34 | rs10805346 | Gout, Serum urate, Uric acid, Serum urate, Urate levels in obese individuals, Urate levels in overweight individuals, Self-reported gout, Treatment with allopurinol |
| 35 | rs6536702 | None |
| 36 | rs871134 | Granulocyte count, Granulocyte percentage of myeloid white cells, Lymphocyte percentage of white cells, Mean platelet volume, Monocyte count, Monocyte percentage of white cells, Myeloid white cell count, Neutrophil count, Neutrophil percentage of white cells, Plateletcrit, Sum basophil neutrophil counts, Sum neutrophil eosinophil counts, White blood cell count, Height |
| 37 | rs4695407 | Height, Arm fat mass right, Body fat percentage, Comparative height size at age 10, Height, Systolic blood pressure, Trunk fat mass, Trunk fat percentage, Weight, Whole body fat mass |
| 38 | rs2282764 | Monocyte count, Height, Arm fat mass left, Trunk fat mass, Weight, Whole body fat mass |
| 39 | rs7705526 | Eosinophil percentage of granulocytes, Eosinophil percentage of white cells, Granulocyte count, Granulocyte percentage of myeloid white cells, Lymphocyte percentage of white cells, Mean corpuscular hemoglobin, Mean corpuscular volume, Mean platelet volume, Myeloid white cell count, Neutrophil count, Neutrophil percentage of granulocytes, Neutrophil percentage of white cells, Platelet count, Platelet distribution width, Plateletcrit, Red blood cell count, Sum basophil neutrophil counts, Sum neutrophil eosinophil counts, White blood cell count, Chronic lymphocytic leukemia, Epithelial ovarian cancer, Granulocyte count, High grade serous ovarian cancer, Invasive epithelial ovarian cancer, Low grade serous and serous borderline ovarian cancer, Lung adenocarcinoma, Lung cancer in never smokers, Myeloid white cell count, Neutrophil count, Plateletcrit, Serous borderline ovarian cancer, Serous invasive ovarian cancer, Sum basophil neutrophil counts, Sum neutrophil eosinophil counts, Telomere length, White blood cell count, Hyperplasia of prostate, Number of operations, Other neoplasms of uncertain or unknown behaviour of lymphoid, haematopoietic and related tissue, Self-reported hypothyroidism or myxoedema, Self-reported polycythaemia vera, Treatment with hydroxycarbamide, Serous boarderline ovarian cancer, High grade serous ovarian cancer, Invasive ovarian cancer, Low grade and borderline serous ovarian cancer, Serous invasive ovarian cancer |
| 40 | rs73730598 | None |
| 41 | rs61748181 | None |
| 42 | rs6881568 | None |
| 43 | rs55747751 | Reticulocyte count, Reticulocyte fraction of red cells, Birth weight, Heel bone mineral density, Self-reported hypertension, Vascular or heart problems diagnosed by doctor: high blood pressure, Vascular or heart problems diagnosed by doctor: none of the above |
| 44 | rs185174247 | None |
| 45 | rs141214782 | None |
| 46 | rs28363070 | Cause of death: thoracic aortic aneurysm, ruptured |
| 47 | rs1611236 | None |
| 48 | rs142730696 | None |
| 49 | rs7772289 | Granulocyte count, Hematocrit, Hemoglobin concentration, Lymphocyte count, Mean corpuscular hemoglobin, Mean corpuscular hemoglobin concentration, Mean corpuscular volume, Monocyte count, Myeloid white cell count, Neutrophil count, Reticulocyte count, Reticulocyte fraction of red cells, Sum basophil neutrophil counts, Sum neutrophil eosinophil counts, White blood cell count, Height, Primary sclerosing cholangitis, Comparative height size at age 10, Diastolic blood pressure, Disorders of mineral metabolism, Forced expiratory volume in 1-second, Forced expiratory volume in 1-second, best measure, Forced vital capacity, Forced vital capacity, best measure, Heel bone mineral density, Intestinal malabsorption, Peak expiratory flow, Potassium in urine, Pulse rate, Risk taking, Self-reported malabsorption or coeliac disease, Self-reported psoriasis, Sitting height, Trunk fat-free mass, Trunk predicted mass, Schizophrenia |
| 50 | rs2763979 | Eosinophil count, Granulocyte count, Hematocrit, Hemoglobin concentration, Lymphocyte count, Monocyte count, Myeloid white cell count, Neutrophil count, Platelet count, Plateletcrit, Red blood cell count, Red cell distribution width, Reticulocyte count, Sum basophil neutrophil counts, Sum eosinophil basophil counts, Sum neutrophil eosinophil counts, White blood cell count, Hypertension, Systolic blood pressure, IgA deficiency, Idiopathic membranous nephropathy, Primary biliary cirrhosis, Rheumatoid arthritis, Type 1 diabetes, Type 1 diabetes combined control dataset gender differentiated, Primary sclerosing cholangitis, Arm fat-free mass left, Arm fat-free mass right, Arm predicted mass left, Arm predicted mass right, Basal metabolic rate, Comparative body size at age 10, Comparative height size at age 10, Doctor diagnosed sarcoidosis, Eye problems or disorders: diabetes related eye disease, Height, Hordeolum and chalazion, Illnesses of siblings: diabetes, Impedance of arm left, Impedance of arm right, Impedance of leg left, Impedance of leg right, Impedance of whole body, Insulin-dependent diabetes mellitus, Intestinal malabsorption, Leg fat-free mass left, Leg fat-free mass right, Leg predicted mass left, Leg predicted mass right, Medication for cholesterol, blood pressure or diabetes: insulin Medication for pain relief, constipation, heartburn: none of the above, Medication for pain relief, constipation, heartburn: paracetamol, Nervous feelings, Self-reported hyperthyroidism or thyrotoxicosis, Self-reported hypothyroidism or myxoedema, Self-reported malabsorption or coeliac disease, Self-reported type 1 diabetes, Sitting height, Started insulin within one year diagnosis of diabetes, Systolic blood pressure, Treatment with carbimazole, Treatment with insulin, Treatment with insulin product, Treatment with levothyroxine sodium, Treatment with paracetamol, Treatment with thyroxine product, Trunk fat-free mass, Trunk predicted mass, Weight, Whole body fat-free mass, Whole body water mass, Rheumatoid arthritis, Rheumatoid arthritis, Rheumatoid arthritis, Schizophrenia, Age at menopause, Diabetes mellitus type 1, Lupus erythematosus systemic |
| 51 | rs80324517 | None |
| 52 | rs201558190 | Lymphocyte count, Rheumatoid arthritis, Rheumatoid arthritis |
| 53 | rs9398196 | Basophil count, Basophil percentage of granulocytes, Basophil percentage of white cells, Granulocyte count, Granulocyte percentage of myeloid white cells, High light scatter percentage of red cells, High light scatter reticulocyte count, Immature fraction of reticulocytes, Lymphocyte percentage of white cells, Mean corpuscular hemoglobin, Mean corpuscular hemoglobin concentration, Mean corpuscular volume, Mean platelet volume, Myeloid white cell count, Neutrophil count, Neutrophil percentage of white cells, Platelet count, Plateletcrit, Red blood cell count, Red cell distribution width, Reticulocyte fraction of red cells, Sum basophil neutrophil counts, Sum eosinophil basophil counts, Sum neutrophil eosinophil counts, White blood cell count, Diastolic blood pressure, Heel bone mineral density, Qualifications: college or university degree, Years of educational attainment |
| 54 | rs61405042 | None |
| 55 | rs2538745 | None |
| 56 | rs117407747 | Eosinophil percentage of granulocytes, Granulocyte count, Granulocyte percentage of myeloid white cells, Monocyte count, Monocyte percentage of white cells, Neutrophil count, Neutrophil percentage of granulocytes, Neutrophil percentage of white cells, Platelet distribution width, Sum basophil neutrophil counts, Sum neutrophil eosinophil counts |
| 57 | rs1985369 | None |
| 58 | rs13230646 | None |
| 59 | rs11769630 | None |
| 60 | rs2056726 | Platelet count, Platelet distribution width, Plateletcrit, Age-related macular degeneration |
| 61 | rs7790856 | None |
| 62 | rs4731541 | Ulcerative colitis, Mouth or teeth dental problems: mouth ulcers, Rheumatoid arthritis |
| 63 | rs117630647 | None |
| 64 | rs2306646 | Mean corpuscular hemoglobin, Mean corpuscular volume, Basal metabolic rate, Height, Hip circumference, Leg fat-free mass left, Leg fat-free mass right, Leg predicted mass left, Leg predicted mass right, Sitting height, Trunk fat mass, Weight, Whole body fat-free mass, Whole body water mass |
| 65 | rs762679 | Eosinophil count, Eosinophil percentage of granulocytes, Eosinophil percentage of white cells, Mean corpuscular hemoglobin, Mean corpuscular volume, Neutrophil percentage of granulocytes, Red blood cell count, Sum eosinophil basophil counts, Mean corpuscular volume, Red blood cell count, Years of educational attainment in females |
| 66 | rs11991877 | None |
| 67 | rs10112752 | Impedance of arm left, Qualifications: college or university degree, Sleep duration |
| 68 | rs1023767 | Body mass index, Arm fat-free mass left, Arm fat-free mass right, Arm predicted mass left, Arm, predicted mass right, Basal metabolic rate, Body mass index, Hip circumference, Impedance of arm left, Impedance of arm right, Impedance of leg left, Impedance of leg right, Impedance of whole body, Leg fat mass left, Leg fat mass right, Leg fat-free mass left, Leg fat-free mass right, Leg predicted mass left, Leg predicted mass right, Pack years adult smoking as proportion of life, span exposed to smoking, Trunk fat-free mass, Trunk predicted mass, Waist circumference, Weight, Whole body fat-free mass, Whole body water mass |
| 69 | rs34896435 | Self-reported testicular cancer |
| 70 | rs11557154 | Granulocyte percentage of myeloid white cells, Immature fraction of reticulocytes, Lymphocyte count, Lymphocyte percentage of white cells, Monocyte count, Monocyte percentage of white cells, Neutrophil percentage of white cells, Body mass index, Body mass index, Neutrophil , ercentage of white cells, Nap during day |
| 71 | rs4743037 | Arm fat-free mass left, Arm fat-free mass right, Arm predicted mass left, Arm predicted mass right, Basal metabolic rate, Comparative height size at age 10, Forced expiratory volume in 1-second, predicted, Height, Leg fat-free mass left, Leg fat-free mass right, Leg predicted mass left, Leg predicted mass right, Sitting height, Trunk fat-free mass, Trunk predicted mass, Whole, body fat-free mass, Whole body water mass |
| 72 | rs117034449 | None |
| 73 | rs7099229 | None |
| 74 | rs12412214 | Granulocyte percentage of myeloid white cells, Mean corpuscular hemoglobin, Mean corpuscular volume, Monocyte count, Monocyte percentage of white cells, Plateletcrit, Red blood cell count, Crohns disease, Inflammatory bowel disease, Ulcerative colitis |
| 75 | rs6584579 | Forced expiratory volume in 1-second, Forced expiratory volume in 1-second, best measure, Forced expiratory volume in 1-second, predicted percentage, Forced vital capacity, Forced vital capacity, best measure, Height |
| 76 | rs9419958 | Height, Leukocyte telomere length, Leukocyte telomere length in females, Telomere length, Atrial fibrillation and flutter, Benign lipomatous neoplasm, Ever had hysterectomy, Number of self-reported cancers, Low grade and borderline serous ovarian cancer |
| 77 | rs77231040 | None |
| 78 | rs10905255 | None |
| 79 | rs939916 | Mean platelet volume, Platelet count, Red cell distribution width, Height |
| 80 | rs10840270 | Bacteremia |
| 81 | rs2293579 | Body mass index females, Body mass index, Chronic kidney disease, Body mass index in males, Body mass index in males, Hip circumference in males, Hip circumference, Waist circumference in males, Waist circumference, Weight, Chronic kidney disease, Serum albumin, Alzheimers disease, log Proinsulin, Serum albumin level, Arm fat mass left, Arm fat mass right, Arm fat percentage left, Arm fat percentage right, Body fat percentage, Comparative body size at age 10, Diastolic blood pressure, Hip circumference, Illnesses of father: high blood pressure, Impedance of leg left, Impedance of leg right, Leg fat mass left, Leg fat-free mass right, Leg predicted mass left, Leg predicted mass right, Medication for cholesterol, blood pressure or diabetes: blood pressure medication, Miserableness, Mood swings, Nervous feelings, Neuroticism score, Self-reported hypertension, Suffer from nerves, Treatment with bendroflumethiazide, Trunk fat mass, Trunk fat percentage, Vascular or heart problems diagnosed by doctor: high blood pressure Vascular or heart problems diagnosed by doctor: none of the above, Waist circumference, Weight, Whole body fat mass, Neuroticism |
| 82 | rs10768683 | None |
| 83 | rs11212631 | None |
| 84 | rs6590343 | Height, Rheumatoid arthritis |
| 85 | rs611646 | Eosinophil count, Granulocyte count, Mean corpuscular hemoglobin, Mean corpuscular volume Myeloid white cell count, Neutrophil count, Platelet count, Plateletcrit, Red blood cell count, Sum basophil neutrophil counts, Sum eosinophil basophil counts, Sum neutrophil eosinophil counts, White blood cell count |
| 86 | rs12369950 | None |
| 87 | rs79977579 | None |
| 88 | rs10773176 | Arm fat mass left, Arm fat mass right, Arm fat percentage left, Arm fat percentage right, Arm fat-free mass left, Arm fat-free mass right, Arm predicted mass left, Arm predicted mass right, Basal metabolic rate, Body fat percentage, Body mass index, Comparative height size at age 10, Forced vital capacity, best measure, Getting up in morning, Height, Hip circumference, Leg fat mass left, Leg fat mass right, Leg fat percentage left, Leg fat percentage right, Leg fat-free mass left, Leg fat-free mass right, Leg predicted mass left, Leg predicted mass right, Pulse rate, Sitting height, Trunk fat mass, Trunk fat percentage, Trunk fat-free mass, Trunk predicted mass, Usual walking pace, Waist circumference, Weight, Whole body fat mass, Whole body fat-free mass, Whole body water mass |
| 89 | rs10845387 | None |
| 90 | rs17445108 | Mean platelet volume, Platelet count, Platelet distribution width |
| 91 | rs10774624 | Basophil count, Eosinophil count, Eosinophil percentage of granulocytes, Eosinophil percentage of white cells, Granulocyte count, Hematocrit, Hemoglobin concentration, High light scatter percentage of red cells, High light scatter reticulocyte count, Immature fraction of reticulocytes, Lymphocyte count, Lymphocyte percentage of white cells, Monocyte count, Myeloid white cell count, Neutrophil count, Neutrophil percentage of granulocytes, Neutrophil percentage of white cells, Platelet count, Platelet distribution width, Plateletcrit, Red blood cell count, Reticulocyte count, Reticulocyte fraction of red cells, Sum basophil neutrophil counts, Sum eosinophil basophil counts, Sum neutrophil eosinophil counts, White blood cell count, Coronary artery disease, Myocardial infarction, Maternal effects on offspring birthweight, Allergic disease, Interferon gamma induced protein 10 levels, Monokine induced by gamma interferon levels, Rheumatoid arthritis, Vitiligo, Acute myocardial infarction, Arm fat-free mass left, Arm fat-free mass right, Arm predicted mass left, Arm predicted mass right, Basal metabolic rate, Birth weight, Birth weight of first child, Chronic ischaemic heart disease, Comparative height size at age 10, Diastolic blood pressure, Doctor diagnosed hayfever or allergic rhinitis, Ever smoked, Hayfever, allergic rhinitis or eczema, Height, Hip circumference, Illnesses of father: heart disease, Illnesses of father: none of the above, group 1, Illnesses of mother: high blood pressure, Illnesses of mother: none of the above, group 1, Illnesses of siblings: high blood pressure, Illnesses of siblings: none of the above, group 1, Impedance of arm left, Impedance of arm right, Impedance of leg left, Impedance of leg right, Impedance of whole body, Leg fat-free mass left, Leg fat-free mass right, Leg predicted mass left, Leg predicted mass right, Mean time to correctly identify matches, Medication for cholesterol, blood pressure or diabetes: blood pressure medication, Medication for cholesterol, blood pressure or diabetes: none of the above, Medication for pain relief, constipation, heartburn: aspirin, Medication for pain relief, constipation, heartburn: omeprazole, No blood clot, bronchitis, emphysema, asthma, rhinitis, eczema or allergy diagnosed by doctor, No treatment with medication for cholesterol, blood pressure, diabetes, or take exogenous hormones, Number of self-reported non-cancer illnesses, Number of treatments or medications taken, Past tobacco smoking, Self-reported gout, Self-reported heart attack or myocardial infarction, Self-reported hypertension, Self-reported hypothyroidism or myxoedema, Self-reported malabsorption or coeliac disease, Self-reported psoriasis, Self-reported stroke, Smoking status: previous, Systolic blood pressure, Taking other prescription medications, Treatment with allopurinol, Treatment with aspirin, Treatment with atenolol, Treatment with bendroflumethiazide, Treatment with blood pressure medication, Treatment with levothyroxine sodium, Treatment with ramipril, Treatment with thyroxine product, Trunk fat-free mass, Trunk predicted mass, Vascular or heart problems diagnosed by doctor: heart attack, Vascular or heart problems diagnosed by doctor: high blood pressure, Vascular or heart problems diagnosed by doctor: none of the above, Vascular or heart problems diagnosed by doctor: stroke, Weight, Whole body fat-free mass, Whole body water mass, Coronary artery disease, Rheumatoid arthritis |
| 92 | rs1907702 | Granulocyte count, High light scatter percentage of red cells, Mean corpuscular hemoglobin, Mean corpuscular volume, Myeloid white cell count, Neutrophil count, Red blood cell count, Sum basophil neutrophil counts, Sum neutrophil eosinophil counts, White blood cell count, Malignant neoplasm of testis, Self-reported testicular cancer |
| 93 | rs76666449 | Morning or evening person |
| 94 | rs28577594 | Lymphocyte count, Lymphocyte percentage of white cells, Neutrophil percentage of white cells, Platelet count, Lymphocyte counts, Arm fat-free mass left, Arm fat-free mass right, Arm predicted mass left, Arm predicted mass right, Basal metabolic rate, Comparative height size at age 10, Diastolic blood pressure, Fluid intelligence score, Height, Leg fat-free mass left, Leg fat-free mass right, Leg predicted mass left, Leg predicted mass right, Nap during day, No blood clot, bronchitis, emphysema, asthma, rhinitis, eczema or allergy diagnosed by doctor, Qualifications: A levels or as levels or equivalent, Qualifications: O levels or GCSEs or equivalent, Qualifications: college or university degree, Qualifications: none, Sitting height, Time spent watching television, Trunk fat-free mass, Trunk predicted mass, Whole body fat-free mass, Whole body water mass, Schizophrenia, Years of educational attainment |
| 95 | rs1332941 | Body mass index in males less than or equal to 50 years of age |
| 96 | rs670180 | Mean corpuscular hemoglobin, Mean corpuscular volume, Platelet count, Plateletcrit |
| 97 | rs9600019 | Hayfever, allergic rhinitis or eczema |
| 98 | rs73581419 | Arm fat-free mass left, Arm fat-free mass right, Arm predicted mass left, Arm predicted mass right, Basal metabolic rate, Height, Leg fat mass left, Leg fat mass right, Sitting height, Trunk fat mass, Trunk fat-free mass, Trunk predicted mass, Weight, Whole body fat mass |
| 99 | rs113525195 | High light scatter percentage of red cells, High light scatter reticulocyte count, Immature fraction of reticulocytes, Mean corpuscular hemoglobin, Mean corpuscular volume, Red cell distribution width, Reticulocyte count, Reticulocyte fraction of red cells, High light scatter reticulocyte count, High light scatter reticulocyte percentage of red cells, Mean corpuscular hemoglobin |
| 100 | rs45604339 | Mean corpuscular hemoglobin, Mean corpuscular hemoglobin concentration, Mean corpuscular volume, Mean platelet volume, Red blood cell count, Arm fat-free mass right, Arm predicted mass right, Basal metabolic rate, Comparative height size at age 10, Height, Sitting height, Sleep duration, Treatment with fluticasone, Trunk fat-free mass, Trunk predicted mass, Whole body fat-free mass, Whole body water mass |
| 101 | rs137901416 | None |
| 102 | rs1957937 | None |
| 103 | rs3093888 | None |
| 104 | rs34550383 | None |
| 105 | rs17677991 | Waist hip ratio adjusted for physical activity in females, Waist hip ratio adjusted for physical activity, Diastolic blood pressure, Impedance of leg right |
| 106 | rs5742915 | Height in females, Height in males, Height, Hip circumference adjusted for BMI, Gene expression, Pagets disease of bone, Pagets disease, Age at first live birth, Age at menarche, Arm fat percentage left, Arm fat percentage right, Arm fat-free mass left, Arm fat-free mass right, Arm predicted mass left, Arm predicted mass right, Basal metabolic rate, Body fat percentage, Body mass index, Comparative height size at age 10, Forced expiratory volume in 1-second, Forced expiratory volume in 1-second, best measure, Forced expiratory volume in 1-second, predicted Forced vital capacity, Forced vital capacity, best measure, Hand grip strength left, Hand grip strength right, Leg fat percentage left, Leg fat percentage right, Number of days or week of vigorous physical activity 10+ minutes, Pulse rate, Sitting height, Sleeplessness or insomnia, Transport type for commuting to job workplace: cycle, Trunk fat-free mass, Trunk predicted mass, Whole body fat-free mass, Whole body water mass, Pagets disease |
| 107 | rs7164950 | Red cell distribution width |
| 108 | rs11412296 | None |
| 109 | rs80116508 | None |
| 110 | rs76219171 | Forced vital capacity, Impedance of arm right, Impedance of whole body, Other malignant neoplasms of skin |
| 111 | rs2967355 | Height, Systolic blood pressure, Arm fat-free mass left, Arm fat-free mass right, Arm predicted mass left, Arm predicted mass right, Basal metabolic rate, Comparative height size at age 10, Forced expiratory volume in 1-second, predicted, Forced vital capacity, Forced vital capacity, best measure, Leg fat-free mass left, Leg fat-free mass right, Leg predicted mass left, Leg predicted mass right, Sitting height, Trunk fat-free mass, Trunk predicted mass, Weight, Whole body fat-free mass, Whole body water mass |
| 112 | rs12925933 | Height |
| 113 | rs111950327 | None |
| 114 | rs450962 | Mean corpuscular hemoglobin, Mean corpuscular volume, Red blood cell count |
| 115 | rs3785074 | Height, Nap during day, Sitting height |
| 116 | rs76065543 | None |
| 117 | rs12932179 | Types of physical activity in last 4 weeks: light diy |
| 118 | rs182059586 | Cause of death: perforation of intestine |
| 119 | rs11117354 | None |
| 120 | rs9940099 | None |
| 121 | rs56061761 | None |
| 122 | rs4724 | None |
| 123 | rs12451892 | Comparative body size at age 10, Diastolic blood pressure, Hair or balding pattern: pattern 4, Systolic blood pressure |
| 124 | rs7209057 | None |
| 125 | rs59409453 | Impedance of arm right |
| 126 | rs111527438 | Arm fat-free mass left, Arm fat-free mass right, Arm predicted mass left, Arm predicted mass right, Basal metabolic rate, Comparative height size at age 10, Height, Leg fat-free mass left, Leg fat-free mass right, Leg predicted mass left, Leg predicted mass right, Sitting height, Trunk fat-free mass, Trunk predicted mass, Weight, Whole body fat-free mass, Whole body water mass |
| 127 | rs7221585 | None |
| 128 | rs75664430 | Ever smoked, Past tobacco smoking, Sleep duration |
| 129 | rs56799554 | Granulocyte percentage of myeloid white cells, Coronary artery disease |
| 130 | rs144204502 | Mean corpuscular hemoglobin, Mean corpuscular volume, Red cell distribution width, Mean corpuscular hemoglobin, Mean corpuscular volume, Red cell distribution width, Treatment with iron sulphate |
| 131 | rs150150565 | None |
| 132 | rs116863223 | None |
| 133 | rs16978028 | Plateletcrit |
| 134 | rs2276182 | Eosinophil percentage of granulocytes, Eosinophil percentage of white cells, Asthma, Hair or balding pattern: pattern 4, Hayfever, allergic rhinitis or eczema, No blood clot, bronchitis, emphysema, asthma, rhinitis, eczema or allergy diagnosed by doctor, Self-reported asthma |
| 135 | rs9955360 | None |
| 136 | rs3891167 | None |
| 137 | rs139669835 | None |
| 138 | rs11085072 | Hematocrit, Hemoglobin concentration, Mean corpuscular hemoglobin, Mean corpuscular volume Red blood cell count, Red cell distribution width, Arm fat-free mass left, Arm fat-free mass right, Arm predicted mass left, Arm predicted mass right, Basal metabolic rate, Impedance of arm left, Impedance of arm right, Impedance of leg right, Impedance of whole body, Leg fat-free mass left, Leg fat-free mass right, Leg predicted mass left, Leg predicted mass right, Peak expiratory flow, Trunk fat-free mass, Trunk predicted mass, Whole body fat-free mass, Whole body water mass |
| 139 | rs4530278 | Basophil count, Basophil percentage of granulocytes, Basophil percentage of white cells, Lymphocyte count, Neutrophil percentage of white cells |
| 140 | rs8105767 | Leukocyte telomere length, Leukocyte telomere length in females |
| 141 | rs429358 | Body mass index, Mean corpuscular hemoglobin concentration, Platelet count, Platelet distribution width, Plateletcrit, Red cell distribution width, Coronary artery disease, Myocardial infarction, Type II diabetes, Age-related macular degeneration, APOE apolipoprotein E, APOE apolipoprotein E females, APOE apolipoprotein E males, Age related macular degeneration, Alzheimers disease, Alzheimers disease age of onset, Cognitive ageing, Cognitive ageing females, Cortical amyloid beta load, LDL cholesterol change with statins, LDL cholesterol female, Late onset Alzheimers disease, Alzheimers disease, Advanced age related macular degeneration, Alzheimers disease biomarkers, Blood protein levels, Brain imaging, Cerebral amyloid deposition PET imaging, Cerebral amyloid deposition positivity PET imaging, Cerebrospinal AB1 42 levels in Alzheimers disease dementia, Cognitive decline age related, Dementia with Lewy bodies, HDL cholesterol, Lewy body disease, Lifespan, Parental lifespan, Platelet count, Red cell distribution width, Arm fat mass left, Arm fat mass right, Arm fat percentage left, Arm fat percentage right, Arm fat-free mass left, Arm predicted mass left, Body fat percentage, Body mass index, Cause of death: alzheimers disease, unspecified, Cause of death: unspecified dementia, Chronic ischaemic heart disease, Diabetes diagnosed by doctor, Diastolic blood pressure, Father still alive, Fathers age at death, Forced expiratory volume in 1-second, best measure, Forced vital capacity, best measure, Frequency of stair climbing in last 4 weeks, Illnesses of father: alzheimers disease or dementia, Illnesses of father: diabetes, Illnesses of father: none of the above, group 1, Illnesses of mother: alzheimers disease or dementia, Illnesses of mother: chronic bronchitis or emphysema, Illnesses of mother: diabetes, Illnesses of mother: high blood pressure, Illnesses of mother: none of the above, group 1, Illnesses of siblings: alzheimers disease or dementia, Leg fat mass left, Leg fat mass right, Leg fat percentage left, Leg fat percentage right, Medication for cholesterol, blood pressure or diabetes: cholesterol lowering medication, Medication for cholesterol, blood pressure or diabetes: none of the above, Mother still alive, Mothers age at death, No treatment with medication for cholesterol, blood pressure, diabetes, or take exogenous hormones, Number of days or week of moderate physical activity 10+ minutes, Number of days or week of vigorous physical activity 10+ minutes, Pain type experienced in last month: back pain, Pulse rate Pulse rate, Self-reported angina, Self-reported dementia or alzheimers/cognitive impairment, Self-reported heart attack or myocardial infarction, Self-reported high cholesterol, Treatment with atorvastatin, Treatment with cholesterol lowering medication, Treatment with ezetimibe, Treatment with ezetrol 10mg tablet, Treatment with lipitor 10mg tablet, Treatment with rosuvastatin, Treatment with simvastatin, Trunk fat mass, Trunk fat percentage, Vascular or heart problems diagnosed by doctor: angina, Vascular or heart problems diagnosed by doctor: heart attack,Waist circumference, Weight, Weight change compared with 1 year ago, Whole body fat mass, Coronary artery disease, C-reactive protein, Low density lipoprotein, Total cholesterol, Posterior cortical atrophy |
| 142 | rs8102497 | None |
| 143 | rs11699829 | Arm fat mass left, Arm fat mass right, Arm fat percentage left, Arm fat percentage right, Body fat percentage, Body mass index, Hip circumference, Leg fat mass left, Leg fat mass right, Leg fat percentage left, Leg fat percentage right, Whole body fat mass |
| 144 | rs142426306 | None |
| 145 | rs1291143 | Red cell distribution width, Alcohol intake frequency, Arm fat-free mass left, Arm fat-free mass right, Arm predicted mass left, Arm predicted mass right, Basal metabolic rate, Comparative height size at age 10, Height, Leg fat-free mass left, Leg fat-free mass right, Leg predicted mass left, Leg predicted mass right, Self-reported fracture thumb, Sitting height, Trunk fat-free mass, Trunk predicted mass, Weight, Whole body fat-free mass, Whole body water mass |
| 146 | rs6054257 | None |
| 147 | rs35640778 | Mean corpuscular hemoglobin, Mean corpuscular volume, Plateletcrit, Cause of death: cerebral infarction, unspecified |
| 148 | rs143190905 | None |
| 149 | rs41304832 | None |
| 150 | rs117512405 | Congenital malformations of cardiac septa |
| 151 | rs28502153 | None |
| 152 | rs6007020 | Hair or balding pattern: pattern 4, Height, Impedance of leg left, Impedance of leg right, Relative age of first facial hair |
| 153 | rs131797 | None |
| 154 | rs1003322 | None |

**Table S3.** MR results using different p-value thresholds for instrument selection.

1. P＜5e-10

| **Method** | **nSNP** | **OR (95% CI)** | **P value** |
| --- | --- | --- | --- |
| Inverse variance weighted | 112 | 0.40 (0.22, 0.75) | 0.004 |
| MR Egger | 112 | 0.21 (0.07, 0.62) | 0.006 |
| Weighted median | 112 | 0.51 (0.18, 1.42) | 0.198 |
| Weighted mode | 112 | 0.48 (0.15, 1.48) | 0.204 |
| Simple mode | 112 | 0.78 (0.09, 6.50) | 0.822 |

1. P＜5e-09

| **Method** | **nSNP** | **OR (95% CI)** | **P value** |
| --- | --- | --- | --- |
| Inverse variance weighted | 119 | 0.39 (0.21, 0.72) | 0.003 |
| MR Egger | 119 | 0.23 (0.08, 0.68) | 0.009 |
| Weighted median | 119 | 0.51 (0.18, 1.40) | 0.190 |
| Weighted mode | 119 | 0.47 (0.15, 1.51) | 0.209 |
| Simple mode | 119 | 0.64 (0.09, 4.66) | 0.659 |

1. P＜5e-07

| **Method** | **nSNP** | **OR (95% CI)** | **P value** |
| --- | --- | --- | --- |
| Inverse variance weighted | 163 | 0.39 (0.21, 0.69) | 0.001 |
| MR Egger | 163 | 0.31 (0.11, 0.86) | 0.026 |
| Weighted median | 163 | 0.51 (0.19, 1.35) | 0.175 |
| Weighted mode | 163 | 0.48 (0.16, 1.44) | 0.190 |
| Simple mode | 163 | 0.32 (0.04, 2.77) | 0.302 |

**Table S4.** Complete MVMR results in current study.

**Table S5.** Univariable MR results for the effects of telomere length on vascular risk factors.

1. Essential hypertension

| **Method** | **nSNP** | **OR (95% CI)** | **P value** |
| --- | --- | --- | --- |
| Inverse variance weighted | 104 | 1.01 (1.01, 1.02) | ＜0.001 |
| MR Egger | 104 | 1.02 (1.01, 1.03) | ＜0.001 |
| Weighted median | 104 | 1.02 (1.01,1.03) | ＜0.001 |
| Weighted mode | 104 | 1.02 (1.01, 1.03) | ＜0.001 |
| Simple mode | 104 | 1.03 (1.00, 1.05) | ＜0.001 |

1. Type 2 diabetes

| **Method** | **nSNP** | **OR (95% CI)** | **P value** |
| --- | --- | --- | --- |
| Inverse variance weighted | 10 | 0.94 (0.68, 1.30) | 0.710 |
| MR Egger | 10 | 1.11 (0.29, 4.23) | 0.883 |
| Weighted median | 10 | 0.93 (0.69, 1.25) | 0.638 |
| Weighted mode | 10 | 1.00 (0.67,1.49) | 0.982 |
| Simple mode | 10 | 1.02 (0.65, 1.61) | 0.935 |

1. Coronary artery disease

| **Method** | **nSNP** | **OR (95% CI)** | **P value** |
| --- | --- | --- | --- |
| Inverse variance weighted | 106 | 0.84 (0.78, 0.90) | ＜0.001 |
| MR Egger | 106 | 0.86 (0.76, 0.98) | 0.022 |
| Weighted median | 106 | 0.87 (0.79, 0.96) | 0.005 |
| Weighted mode | 106 | 0.86 (0.78, 0.95) | 0.003 |
| Simple mode | 106 | 0.80 (0.66, 0.97) | 0.026 |

**Table S6.** Percentage of comorbid patients in normal pressure hydrocephalus (NPH) cases.

| **Diseases** | **Percentage (%)** | **Diagnostic criteria** |
| --- | --- | --- |
| **Disorders with the same clinical symptoms** |  |  |
| Vascular dementia | 4.01 | ICD-10: F01 |
| Parkinson’s disease | 6.84 | ICD-10: G20 |
| Alzheimer’s disease | 16.94 | ICD-10: G30 |
| Unspecified dementia | 8.92 | ICD-10: F03 |
| Ischaemic stroke | 19.32 | ICD-10: I63; I64 |
| Transient ischemic attack | 14.12 | ICD-10: G45 |
| **Etiology of secondary NPH** |  |  |
| Subarachnoid haemorrhage | 2.97 | ICD-10: I60 |
| Intracerebral haemorrhage | 4.46 | ICD-10: I61 |
| Other intracranial haemorrhage | 3.86 | ICD-10: I62 |
| Ischaemic stroke | 19.32 | ICD-10: I63; I64 |
| Traumatic brain injury | 10.25 | ICD-10: S06.2; S06.3; S06.4; S06.5; S06.6; S06.7 |
| Malignant neoplasm of brain | 1.63 | ICD-10: C71 |
| Malignant neoplasm of meninges | 1.19 | ICD-10: C70 |
| Meningitis | 1.19 | ICD-10: G0; G1 |

**Figure S1.** Sensitivity analysis for the association between telomere length and iNPH. **(A)** Funnel plot **(B)** Leave-one-out analysis.


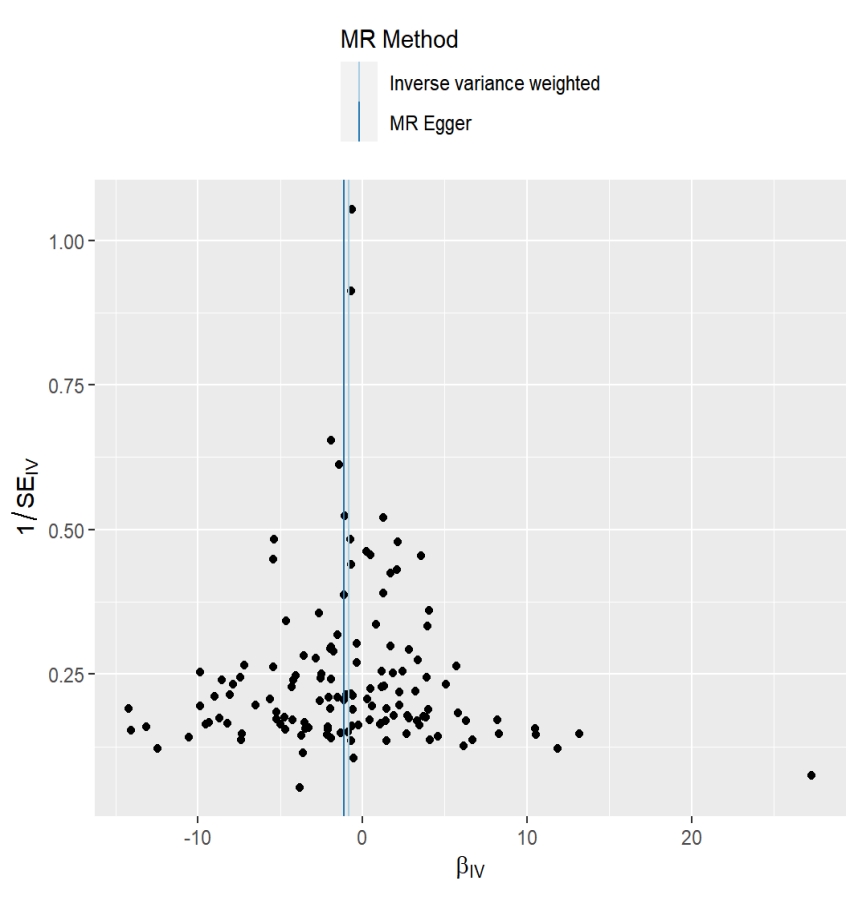


**(A)**


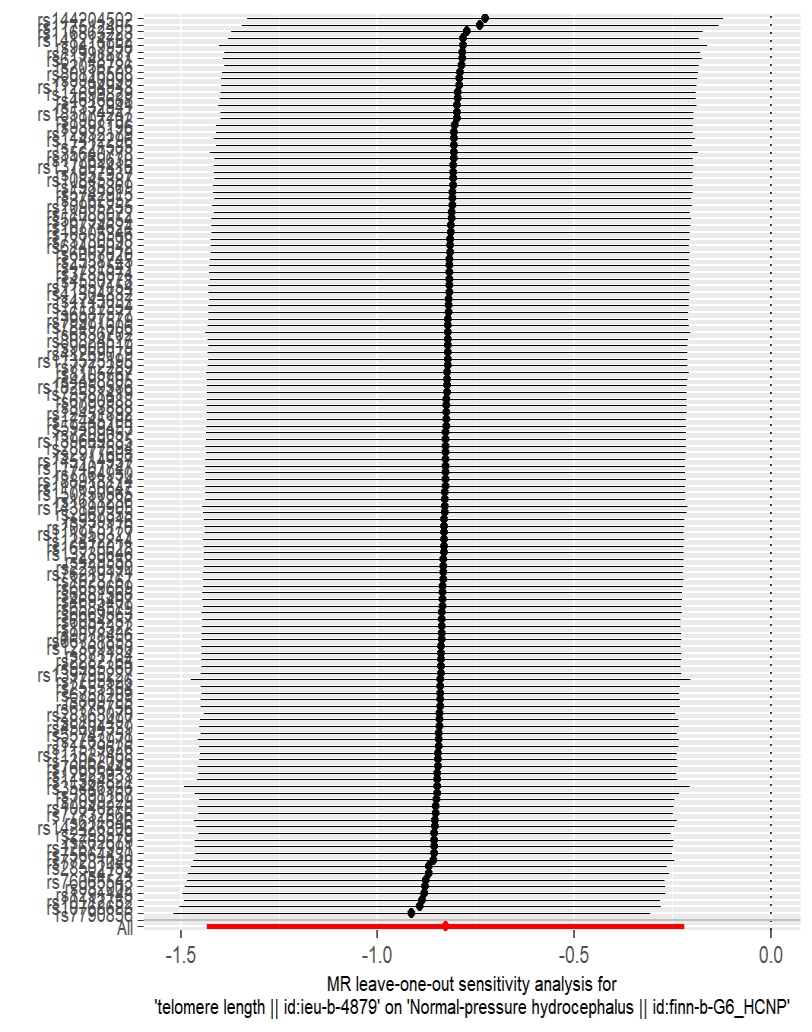


**(B)**
